# Gastrointestinal Manifestations of COVID-19 Infection: Clinicopathologic Findings in Intestinal Resections Performed at Single Institution

**DOI:** 10.3389/fmed.2022.811546

**Published:** 2022-02-14

**Authors:** Alison E. Burkett, Sophia B. Sher, Chirag R. Patel, Isam Ildin-Eltoum, Deepti Dhall, Camilla Margaroli, Shajan Peter, Goo Lee, Prachi Bajpai, Paul V. Benson, Upender Manne, Sameer Al Diffalha

**Affiliations:** ^1^Department of Pathology, University of Alabama at Birmingham, Birmingham, AL, United States; ^2^Division of Pulmonary, Allergy and Critical Care Medicine, Department of Medicine, University of Alabama at Birmingham, Birmingham, AL, United States; ^3^Division of Gastroenterology, Department of Medicine, University of Alabama at Birmingham, Birmingham, AL, United States; ^4^O'Neal Compressive Cancer Center, Birmingham, AL, United States

**Keywords:** COVID-19, gastrointestinal manifestations, pneumatosis cystoides intestinalis, ischemic colitis, ISH

## Abstract

It is now known that COVID-19 not only involves the lungs, but other organs as well including the gastrointestinal tract. Although clinic-pathological features are well-described in lungs, the histopathologic features of gastrointestinal involvement in resection specimens are not well characterized. Herein, we describe in detail the clinicopathologic features of intestinal resection specimens in four patients with COVID-19 infection. COVID-19 viral particles by *in situ* hybridization and immunofluorescence studies are also demonstrated. All four patients were males, aged 28–46 years, with comorbidities. They initially presented with a severe form of pulmonary COVID-19 and showed gastrointestinal symptoms, requiring surgical intervention. Histopathologic examination of resected GI specimens, mostly right colectomies, revealed a spectrum of disease, from superficial mucosal ischemic colitis to frank transmural ischemic colitis and associated changes consistent with pneumatosis cystoides intestinalis. Three patients were African American (75%), and one was Caucasian (25%); three patients died due to complications of their COVID-19 infection (75%), while one ultimately recovered from their GI complications (25%), but experienced prolonged sequela of COVID-19 infection including erectile dysfunction. In conclusion, COVID-19 infection, directly or indirectly, can cause ischemic gastrointestinal complications, with predilection for the right colon.

## Introduction

In December 2019, a new human coronavirus (SARS-CoV-2) type emerged in Wuhan, China. COVID-19, the infectious disease caused by SARS-CoV-2, is now a pandemic affecting countries throughout the world. Since spring 2020, the United States has seen a dramatic rise in number of cases, and currently the number of deaths attributable to COVID-19 infection is more than 500,000 (1).

Although the most common presentation of the infection is the development of respiratory symptoms 2–14 days following exposure, gastrointestinal (GI) presentation is becoming increasingly recognized. The receptor of SARS-CoV-2, angiotensin converting enzyme 2 (ACE2) is highly expressed both in GI epithelial cells and in liver (2). Almost the entire GI tract, including the stomach, small intestine, and rectal epithelial cells express the SARS-CoV-2 nucleocapsid protein and the ACE2 protein (3). The SARS-CoV-2 nucleocapsid protein, which encapsulates the viral genome, is essential for SARS-CoV-2 replication (4). High levels of these two proteins in cells of the GI tracts of SARS-CoV-2 infected patients can explain the concomitant digestive symptoms, including diarrhea, nausea, vomiting, and abdominal pain (3).

Here, we present a series of four SARS-CoV-2 patients who were admitted to the University of Alabama at Birmingham (UAB) Medical Center during June to August 2020 and who underwent GI resections. The course of their SARS- CoV-2 infections was remarkable for the development of GI complications, which necessitated surgical management by right hemicolectomy or segmental small bowel resection; three of the four patients ultimately died as a result of the complications of their COVID-19 infection. The clinical, macroscopic and histopathologic findings are described, which adds to the pathophysiology of SARS-CoV-2 infection and contributes to the ongoing management of the COVID-19 disease.

## Materials and Methods

The Institutional Review Board of UAB approved the study. A retrospective review of surgical pathology archives for COVID-19 related intestinal resections was performed during the period of June to August 2020. There were four patients with bowel resections, three of whom underwent right hemicolectomies and one a segmental small bowel resection. The hematoxylin and eosin (H&E)-stained slides were reviewed by GI pathologists (SA, IE, DD, and CRP).

In this study, for detection of SARS-CoV2 virus, both immunofluorescence and *in situ* hybridization (ISH) assays were performed as follows: 5-μm formalin-fixed paraffin-embedded (FFPE) tissues sections on Plus Slides (VWR, Radnor, PA) were cut at 5 μm and baked for 2 h at 60°C. Tissues were then deparaffinized and rehydrated with sequential 5-min incubations in xylene (three times), twice in 100% ethanol, and twice in 95% ethanol. Slides were washed three times, for 5 min each, with distilled water. Antigen retrieval was then performed in Tris-EDTA pH-9 buffer at 70°C for 20 min in a steamer, followed by three 5-min washes in distilled water. Tissues were balanced with PBS for 10 min and blocked with 3% w/v bovine serum albumen (BSA) in PBS for 40 min at room temperature. Slides were then blotted dry, and antibody against SARS-CoV-2 nucleocapsid protein (clone GTX135361, GeneTex, Irvine, CA) was applied at a 1:500 dilution in PBS+3% w/v BSA for 1 h at room temperature. Tissues were then rinsed with PBS three times for 5 min under gentle agitation. 4′,6-Diamidino-2-phenylindole (100 ng/mL in PBS) staining was performed for 5 min in the dark at room temperature, followed by three 5-min washes in PBS under gentle agitation. Slides were then mounted with ProLong Gold antifade mounting media (Thermofisher) and stored in the dark until image acquisition. Confocal immunofluorescence images were acquired with a Nikon A1R confocal microscope.

ISH assay was performed using RNAscope® kit and ISH probes according to the manufacturer's instructions (Advanced Cell Diagnostics, ACD, Newark, CA). RNA probe, in C2 channel targeting SARS-CoV-2 replicative RNA intermediate, was used to detect the replicating virus as red signal (nCoV2019-orf1ab-sense-C2, cat. no. 859151-C2). RNAscope® 2.5 Duplex Reagent Kit (cat. no 322430) along with Human (Hs) Positive Control Probes for housekeeping genes PPIB-C1/ POLR2A-C2, (cat. no 321641) were used to assess the integrity of the RNA. Simultaneously, consecutive sections were probed with probes targeting dihydrodipicolinate reductase B mRNA of a *Bacillus subtilis* strain (DapB) as a negative control, (cat. no. 320751) to assess the specificity of the assay.

## Results

### Clinical Data

Our cohort included four patients aged 28–46 years with a mean age of 38 years. Three of four patients were African-American (AA); one was non-Hispanic Caucasian. All patients had comorbidities that included essential hypertension (HTN), diabetes mellitus (DM, insulin-dependent or insulin-resistant), and obesity (BMI 33.08–34.8 kg/m^2^). All patients initially presented with respiratory symptoms, including fever, dyspnea, and/or flu-like symptoms which developed into acute respiratory distress syndrome (ARDS) requiring intubation, ventilation, and critical care management. The length of time between a positive COVID-19 test and significant GI symptomatology and resection varied widely from 1 to 53 days, with an average of 21.5 days. GI symptoms occurred on a spectrum, beginning as constipation, abdominal pain, or a lower GI bleed before progressing to ischemia and necrosis requiring surgical intervention. COVID-19 infections were confirmed by the presence of SARS-CoV-2 RNA, determined by PCR. The patients were enrolled in a clinical trial of remdesivir. Despite the treatment, they subsequently developed complications, such as lower extremity deep vein thrombosis, pulmonary thromboembolism, septicemia with septic shock (due to *Cutibacterium/Propionibacterium avidum*), multi-organ failure, hypotension, pulseless electrical activity and encephalopathy. For all four patients, acute renal failure was a complication, and all underwent continuous renal replacement therapy. Furthermore, the course of their disease was complicated by the development of GI symptoms, such as GI bleeding in the form of hematochezia, as well as an ileus. All patients underwent CT scans of the abdomen and pelvis, which showed changes compatible with GI bleeding with various degrees of intestinal pneumatosis and bowel ischemia. These findings were corroborated by the frank ischemia, ulceration, cecal dilation, and necrosis encountered intraoperatively. Additionally, laboratory findings were remarkable for elevated C-reactive protein (ranging from 24.87 to 282.6 mg/L), d-dimer (ranging from 978 to > 20,000 ng/mL), lactate dehydrogenase (556–681 U/L), lactic acid (4.0–10.6 mMol/L), and troponin I (39–1838 ng/L). Most patients were also anemic (Hgb between 7.3 and 11 g/dL; Hct 22–33%). The three patients who expired due to the infection demonstrated the most dramatic clinical courses, with pronounced laboratory, gross, and microscopic abnormalities (Table 1). For all four patients with SARS-CoV-2 infection confirmed by nasal swap PCR testing, there was colonic pneumatosis with or without ischemic changes for whom CT scans of the abdomen showed evidence of gas in the mesentery, or features suggestive of lower GI bleeding.

**Table 1:** Patients' demographic, laboratory values and pathologic findings.

	**Demographics**				**Laboratory values**						**Pathologic findings**	
**Case #**	**Age**	**Sex**	**Race**	**Comorbidities**	**CRP (mg/L)**	**D-dimer (ng/ml)**	**LDH (Units/L)**	**Lactic acid (mMol/L)**	**Troponin I (ng/L)**	**Hgb/Hct**	**Gross findings**	**Histology**
Case 1	47	M	AA	HTN, Obesity (BMI 33.08 kg/m^2^)	176.3	4,064	NA	4.0	39	9.9/26	Large patches of erythematous colonic mucosa with focal loss of mucosal folds.	Ischemic colitis showing transmural acute and chronic inflammation and serositis. Features suggestive of pneumatosis.
Case 2	37	M	AA	HTN, Obesity, Insulin Dependent DM	282.6	978	556	NA	44	11/33	Cecum showed several areas of ulceration ranging in size from 0.6 to 1.0 cm	Focal mucosal ulceration, with associated acute and chronic inflammation. Features suggestive of pneumatosis. Focal serositis.
Case 3	40	M	C	Obesity (BMI 33.2 kg/m^2^)	NA	>20,000	NA	7.1	1,838	15.6/46	Cecum: Necrotic with dark-brown discoloration. Ascending colon is hemorrhagic and dusky with obvious air bubbles	Mucosal hemorrhagic ischemia, fibrin microthrombi, and pneumatosis cystoides intestinalis
Case 4	28	M	AA	DM	24.87	1,380	681	10.6	98	7.3/22	Small intestine: Granular/gritty brown mucosa with diffuse gray-brown exudate	Transmural ischemia with pseudomembrane formation and acute serositis. Features suggestive of pneumatosis.

### Gross and Microscopic Characteristics

**Figure 1:**
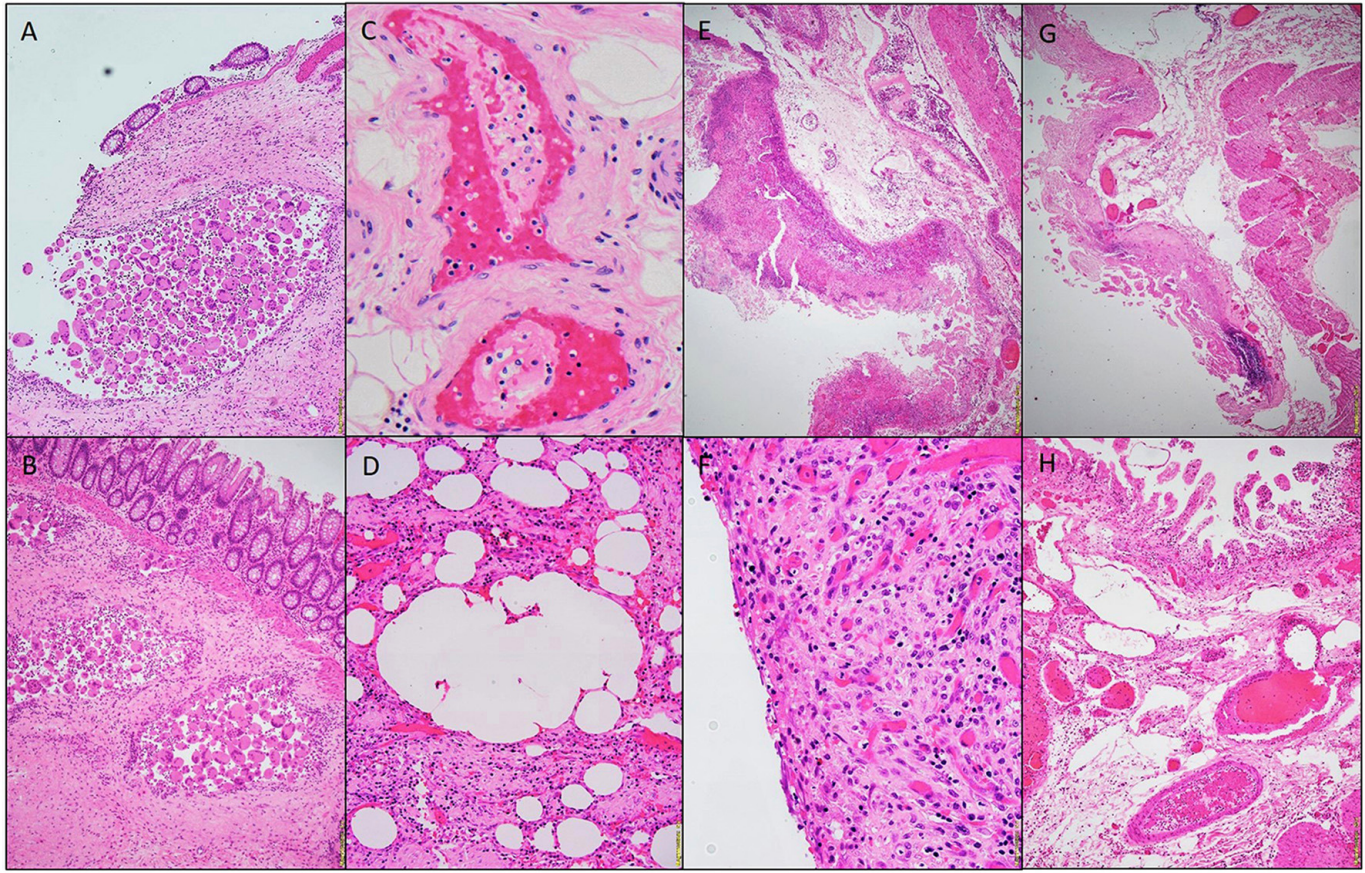
Photomicrographs of a hemicolectomy specimen. Aggregates of multinucleated giant cells lining the cysts. H&E stain 20x **(A,B)**. Fibrin thrombi, H&E staining 40x **(C)**. Micro-cyst lined with multinucleated giant cells, H&E staining 10x **(D)**. Pseudomembrane, H&E 10x **(E)**. Acute serositis, H&E staining 40x **(F)**. Ischemic enteritis, H&E 10x **(G)**. Submucosal thrombi, H&E 10x **(H)**.

**Figure 2:**
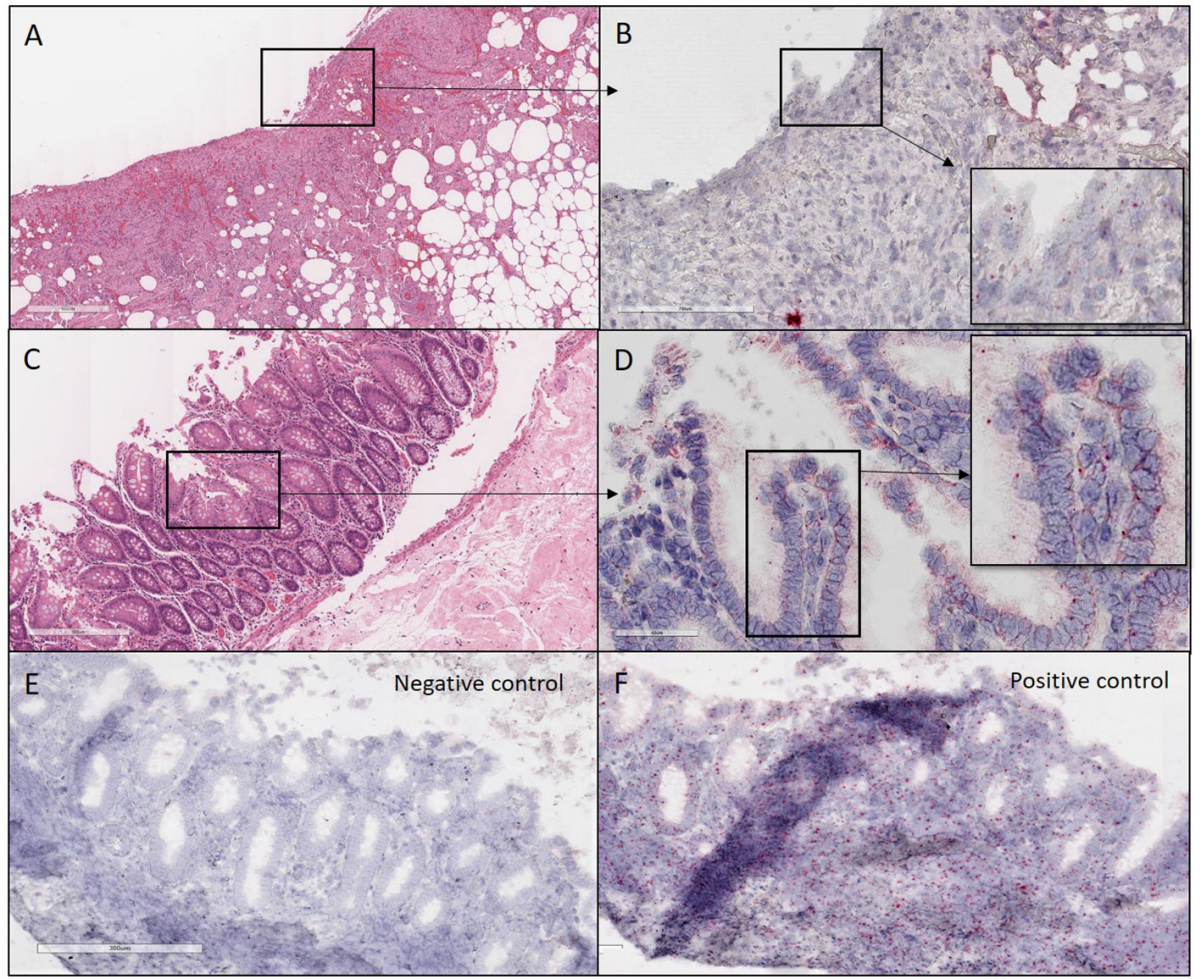
H&E and ISH images of patient #1 in focal mucosal ulcer with granulation tissue formation along with adjacent uninvolved mucosa. **(A)** H&E images of ulcerated colon tissue at 4X magnification **(B)** Images at 20X magnification after *in situ* hybridization using V-nCOV2019—orf1ab–sense probe (cat # 859151-C2), ACD, Bio-techne, detecting replicating SARS-COV-2 RNA (red chromogen) in ulcerated colon tissue sections. **(C)** H&E images at 10x magnification of adjacent uninvolved mucosa **(D)**. Higher magnification images at 40X after *in situ* hybridization using V-nCOV2019—orf1ab-detected as red signal in adjacent uninvolved mucosa. **(E)** Negative control images at 10X after *in situ* hybridization using probe (cat # 320751), ACD, Bio-techne, targeting DapB (Bacillus subtilis stain) which did not detect any staining **(F)**. Positive control images at 10X after *in situ* hybridization using probe (cat # 321641), ACD, Bio-techne, demonstrated strong positive staining (red chromogen) in sections probed for the housekeeping gene POLR2A.

**Figure 3:**
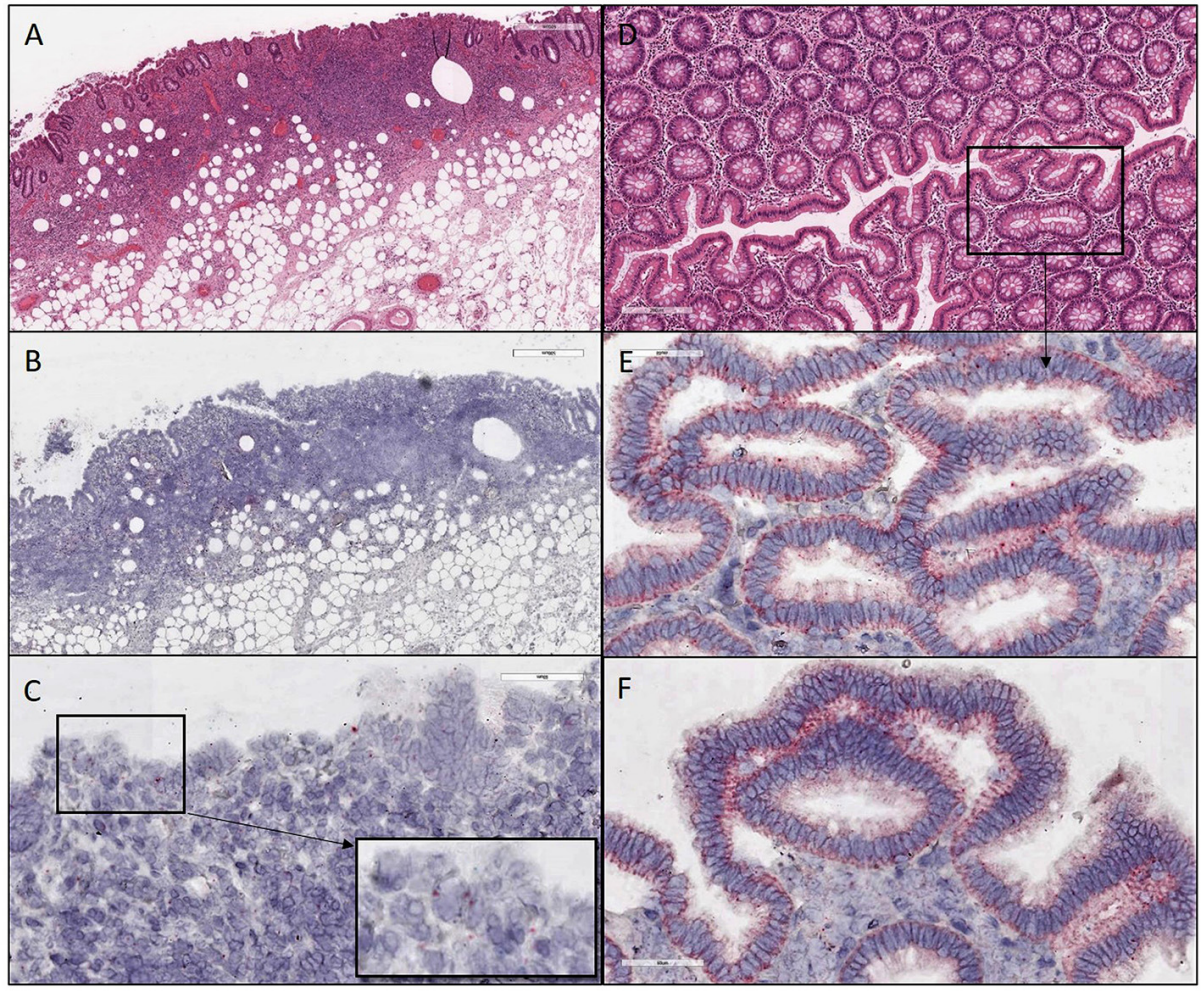
H&E and ISH images of patient #2 in focal mucosal ulcer with granulation tissue formation along with adjacent uninvolved mucosa. **(A)** H&E images of ulcerated colon tissue at 4X magnification **(B,C)** Images after *in situ* hybridization using V-nCOV2019—orf1ab–sense probe (red chromogen) at lower 4X and higher magnification 40x, respectively. **(D)** H&E images at 10X magnification of adjacent uninvolved mucosa **(E,F)** *in situ* hybridization using V-nCOV2019—orf1ab- sense probe detected as red signal in adjacent intact epithelium of uninvolved mucosa at higher magnification images at 40x. Images show pronounced viral load in the surface epithelial lining and the crypts (Basal cytoplasmic localization).

**Figure 4:**
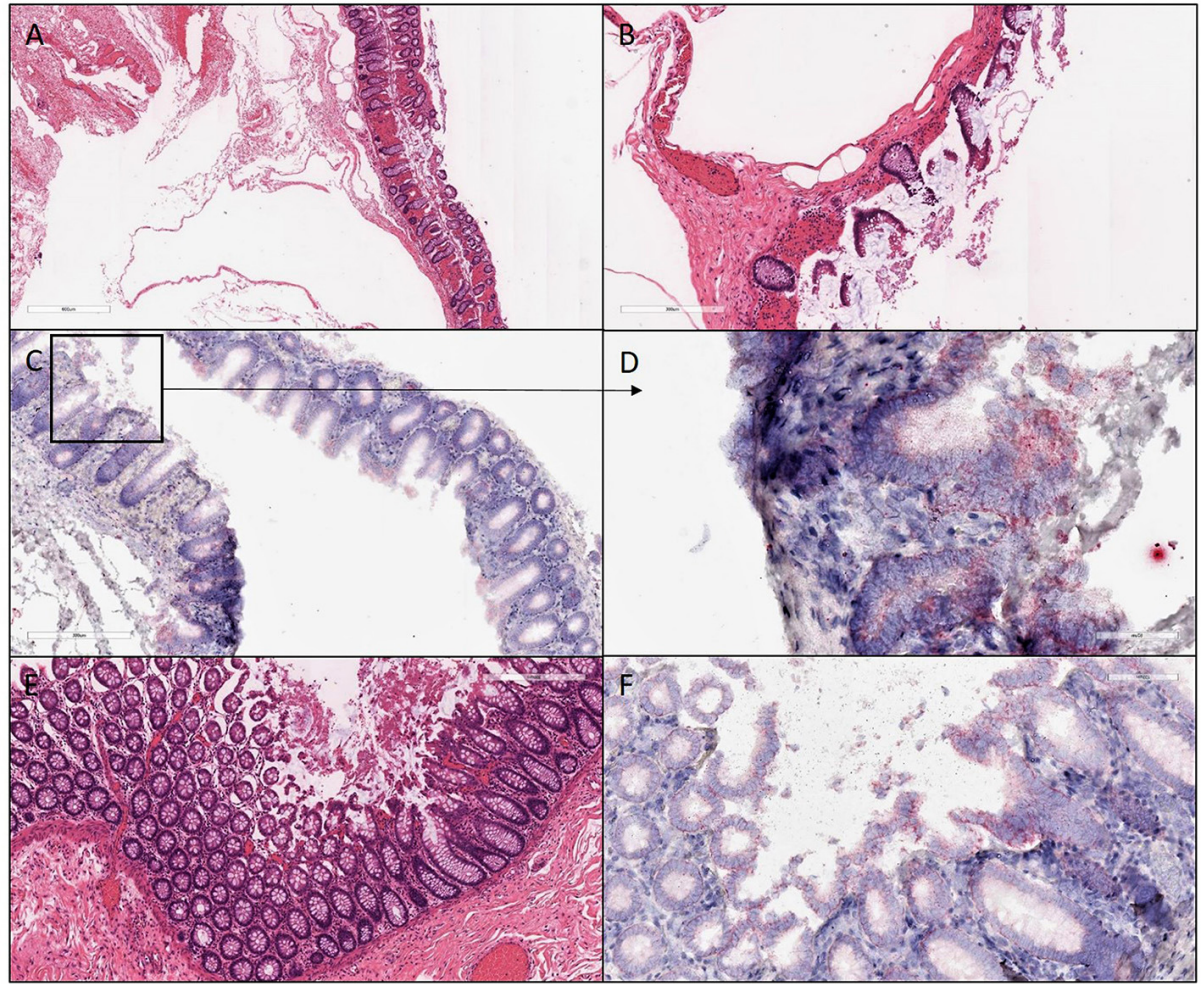
H&E and ISH images of patient #3 in focal mucosal ulcer with granulation tissue formation along with adjacent uninvolved mucosa. **(A,B)** H&E images of ischemic colon tissue sections at 4X and 10X magnification, respectively. **(C,D)** Images at lower (10X) and higher magnification (40X), respectively, after *in situ* hybridization using V-nCOV2019—orf1ab–sense probe (red chromogen) More viral load of replicating SARS-COV-2 RNA was detected in this ischemic section as compared to its uninvolved mucosa represented in **(F)**. **(E)** H&E images at 10X of adjacent uninvolved mucosa **(F)** i*n situ* hybridization using V-nCOV2019—orf1ab- sense probe detected as red signal in adjacent intact epithelium of uninvolved mucosa at 20X magnification.

**Figure 5:**
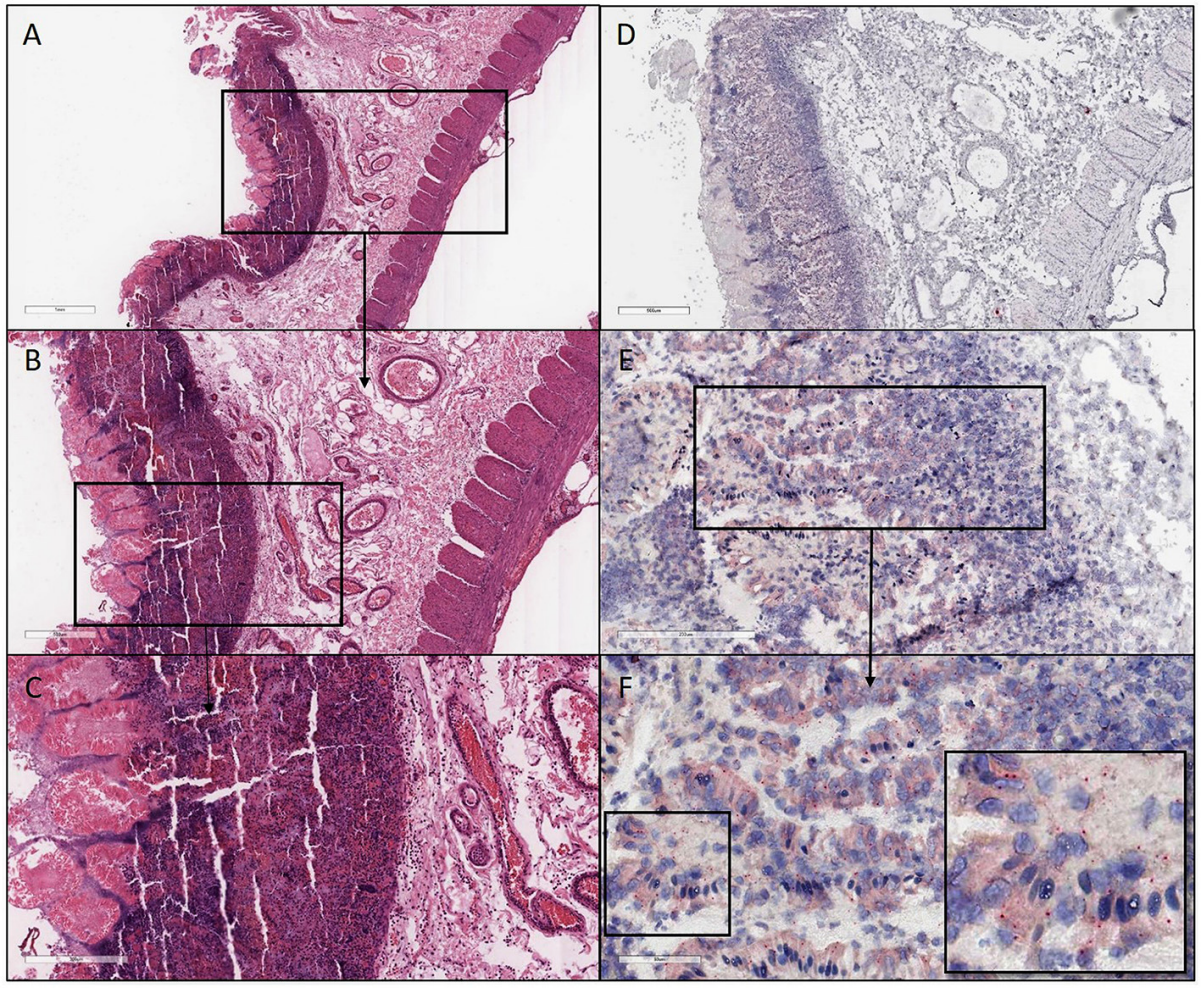
H&E and ISH images of patient #4 in focal mucosal ulcer with granulation tissue formation. **(A–C)** H&E images of ischemic colon tissue sections at 2X, 4X, and 10X magnification, respectively. **(D–F)** Images at lower (4X) and higher magnification (20X and 40X), respectively, after *in situ* hybridization using V-nCOV2019—orf1ab–sense probe (red chromogen) illustrating replicating SARS-COV-2 RNA was detected.

Three patients had right hemicolectomies and one had a segmental small bowel resection. All four specimens showed variable degrees of ischemic changes. Gross examination of one of sample showed transmural ischemic necrosis with hemorrhage, resulting in perforation. The mucosa showed, microscopically, deep ulceration and focal pseudomembrane formation. In an additional patient, there was diffuse transmural acute and chronic inflammation with necrosis and hemorrhage, and associated acute serositis. The lamina propria demonstrated congestion and edema (in all patients); rare vascular fibrin thrombi were identified (in 2 patients). Pneumatosis cystoides intestinalis (cyst formation with associated multinucleated giant cells lining) were also recognized in two patients (Figures 1–5 and Table 1).

ISH with a V-nCOV2019-sense probe detected replicating SARS-COV-2 RNA. Furthermore, all four samples were positive on immunofluorescence for antibody against SARS-CoV-2 nucleocapsid protein with a distinctive pattern and a variable viral load (Figures 2–4). All patients underwent exploratory laparotomies for GI complications of COVID-19 disease, and three of the four patients underwent a right hemicolectomy procedure. The morphologic findings in all specimens included ischemic changes, acute and chronic inflammation, and fibrin microthrombi in small blood vessels in underlying areas of mucosal ulceration. Moreover, all cases, regardless of extent of the disease, showed changes consistent with pneumatosis cystoides intestinalis, including prominent submucosal edema with occasional cyst formation and aggregates of multinucleated giant cells.

## Discussion

**Figure 6:**
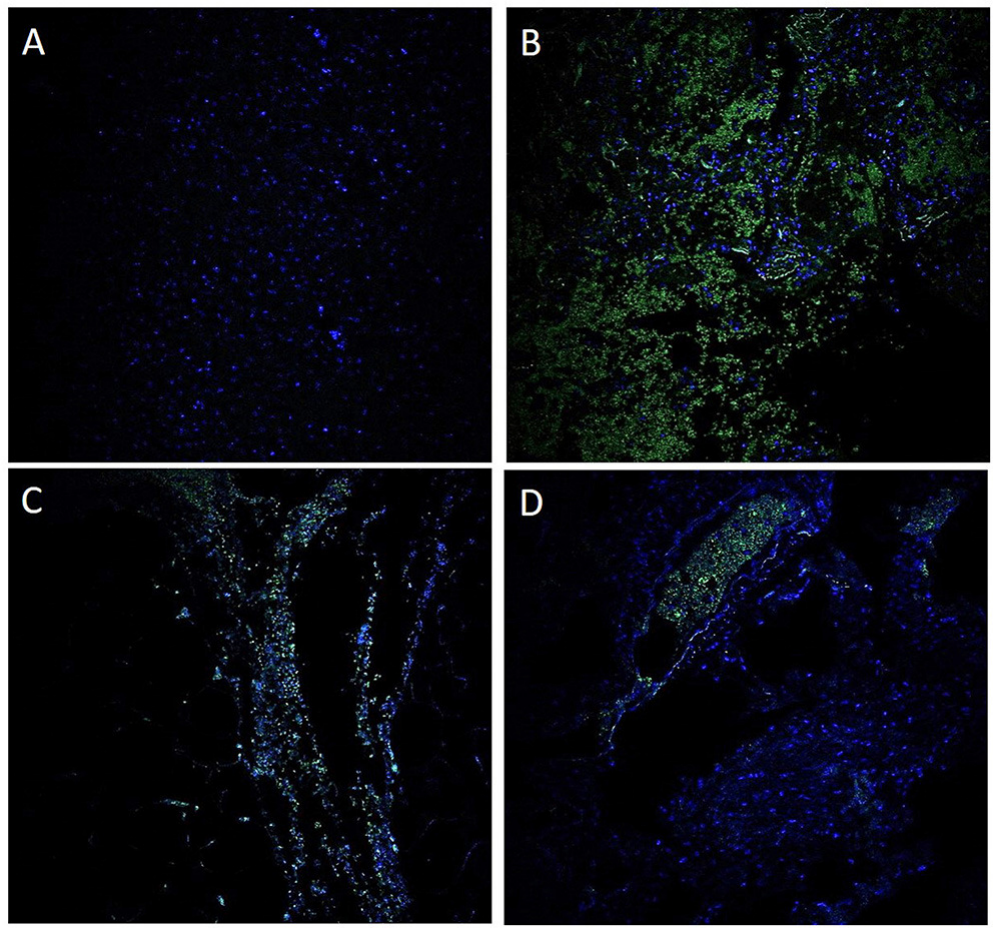
Immunofluorescence for antibody against SARS-CoV-2 nucleocapsid protein. A negative control using a probe targeting DapB (Bacillus subtilis strain) did not detect any staining **(A)**; a positive control demonstrated strong positive staining in sections probed with the housekeeping gene **(B)**. These findings suggest that the GI tract is infected with SARS-CoV-2 **(C)**. A unique observation in our case study is the presence of the virus within the blood vessels **(D)**.

GI symptoms associated with COVID-19 are present in up to 30% of patients, with diarrhea, abdominal pain, and hematochezia occasionally evident as the initial presentation (5). Although patients with significant pulmonary disease have detectable SARS-CoV-2 RNA in fecal samples, a substantial number of patients with GI manifestations also have SARS-CoV-2 RNA in fecal samples (6). Others have now individualistically established that the virus can be cultured from the feces during an active infection (7). In the present study, we demonstrated by immunofluorescence (Figure 6) and ISH studies (Figures 2–5) evidence of the virus in affected area of the intestine.

Notably, neither of the patients had a medical history of established GI disease, nor etiology for colitis. Extensive infectious work up including *Clostridium difficile* serology was conducted on two of the four patients, which was negative. Reactivity for CMV IgG was noted in the serum of one patient indicating a previous infection; however, no evidence of viral cytopathic effects were noted on histology. One patient had clinical and microbiological evidence of septicemia due to *Cutibacterium (Propionibacterium) avidum*, the commensal skin microorganism, which is unlikely to be implicated as a primary agent in the etiology of hemorrhagic colitis. One patient developed HIT following anticoagulation, resulting in septic shock. While this further exacerbated the bowel ischemia and thrombosis, it was a contributing factor rather than a primary cause. Moreover, extensive chart review failed to reveal definitive evidence of mesenteric thrombosis, radiologically or intraoperatively, to explain the segmental ischemic findings.

The target viral receptor for SARS-CoV-2 is ACE2, which is highly expressed on type II alveolar epithelial cells, as well as in glandular cells of gastric, duodenal, and rectal epithelia (8). This suggests that the SARS-CoV-2 gains entry into, and potentially damages, host GI tissue, explaining the digestive symptoms. Microthrombi also contribute to GI insults (9).

Although several studies have attempted to identify histologic findings indicative of SARS-CoV-2 infection, the presentation and expression of SARS-CoV-2 RNA remains variable (10). In our cohort, not all GI specimens demonstrated findings of coagulopathy and ischemia, but all samples revealed changes consistent with pneumatosis cystoides intestinalis. While pneumatosis cystoides is commonly associated with bacterial infection, its pathogenesis is poorly understood and multifocal, and can also be attributed to mechanical and pulmonary causes (11). In our cohort, those patients that underwent infectious work-up for gas producing bacteria were negative. Moreover, these patients were acutely ill, requiring repeated surgical intervention, intubation and ventilation. This suggested that the etiology of pneumatosis could be the result of iatrogenic trauma or manipulation increasing intraluminal pressure (11), or the result of free air entering the perivascular spaces of the intestinal wall following alveolar rupture in the setting of pulmonary obstruction (12); the clinical significance failed to be elucidated. The pathogenic pathway of COVID-19 remains largely undetermined, and its relationship with the gastrointestinal findings remains a challenge. It is widely accepted that the viral infection results in microthrombi which can lead to ischemic injury; however, in the setting of critical illness, HIT, and intragenic interventions (ECMO) it is difficult to quantify the exact role SARS-CoV-2 virus plays. The clinicopathologic findings in our cohort caused us to consider the relationship between the ischemic enterocolitis and hypercoaguable state of our patients with COVID-19 infection; however, comparison with non-COVID associated acute ischemic bowels should be performed to further characterize and ultimately define the histopathological features of the virus.

Moreover, each GI specimen demonstrated SARS-CoV-2 positivity via ISH, regardless of the extent of disease. Coronaviruses are also found by electron microscopy in intestinal cells of animals (13). Comparable to our study, SARS-CoV-2 RNA ISH is reported to be positive in a few mucosal epithelial cells and lymphocytes of the GI tract in humans (7). Also what supports our findings is that SARS-Cov-2 RNA in stool, determined by amplification is now widely accepted (7).

In a recent study that included a large number of SARS-CoV-2 patients (n=95), 58 patients experienced GI symptoms including diarrhea, anorexia and nausea (14). Fecal samples of 65 hospitalized patients were tested for SARS-CoV-2, including 42 patients with and 23 without GI symptoms, of which 22 (52.4%) and 9(39.1%) were positive, respectively. Six of the patients who had GI symptoms underwent endoscopic examination; one showed erosion/ulcer. In two, SARS-CoV-2 RNA was detected in the esophagus, stomach, duodenum, and rectum (14).

In the present study, there were pathologic findings for the four patients with confirmed SARS-CoV-2, for whom CT scans of the abdomen showed either evidence of colonic pneumatosis with or without ischemic changes and evidence of gas in the mesentery, or features suggestive of lower GI bleeding. All four patients underwent exploratory laparotomies and three of the four were subjected to a right hemicolectomy procedure for COVID-19 GI related complications. The presence of microthrombi in the watershed area suggested that SARS-CoV-2 demonstrates partiality toward the right colon. Moreover, all cases, regardless of extent of the disease, showed pathologic changes consistent with pneumatosis cystoides intestinalis.

For some patients, GI symptoms may be associated with pulmonary SARS-CoV-2 infection, and surgical management of GI complications, including bleeding, is sometimes necessary. The morphologic findings observed in the resection specimens varied from superficial acute colitis with or without hemorrhage and ischemic changes, to a frank transmural necrotic colitis with fibrin microthrombi. Changes consistent with pneumatosis cystoides intestinalis were also evident. In conclusion, COVID-19 disease, directly or indirectly, can cause ischemic GI complications, with a predilection for the right colon. Awareness of these morphologic changes may prompt pathologists to include potential SARS-CoV-2 infection to the differential diagnosis when the etiology for ischemic colitis is unclear.

## Data Availability Statement

The original contributions presented in the study are included in the article/supplementary material, further inquiries can be directed to the corresponding author.

## Ethics Statement

Ethical review and approval was not required for the study on human participants in accordance with the local legislation and institutional requirements. Written informed consent for participation was not required for this study in accordance with the national legislation and the institutional requirements.

## Author Contributions

SA principal investigator and corresponding author, conceptualized the study with SS and AB, finalized the manuscript with the assistance of AB. CM, PB, and UM performed the *in-situ* Hybridization and reviewed the results and critically reviewed the manuscript. SP reviewed and revised the manuscript. CP, II, DD, GL, and PVB, contributed in data collection. All authors approved the final manuscript as submitted and agree to be accountable for all aspects of the work.

## Funding

This work is supported, in part, by the institutional funds to SA and UM.

## Conflict of Interest

The authors declare that the research was conducted in the absence of any commercial or financial relationships that could be construed as a potential conflict of interest.

## Publisher's Note

All claims expressed in this article are solely those of the authors and do not necessarily represent those of their affiliated organizations, or those of the publisher, the editors and the reviewers. Any product that may be evaluated in this article, or claim that may be made by its manufacturer, is not guaranteed or endorsed by the publisher.
